# Supplementary figures and images for: Exploiting peptide chirality and transport to dissect the complex mechanism of action of host peptides on bacteria
Source: PLoS Genet. 2025 Dec 11;21(12):e1011892. doi: 10.1371/journal.pgen.1011892 (PMC12714254; doi:10.1371/journal.pgen.1011892)

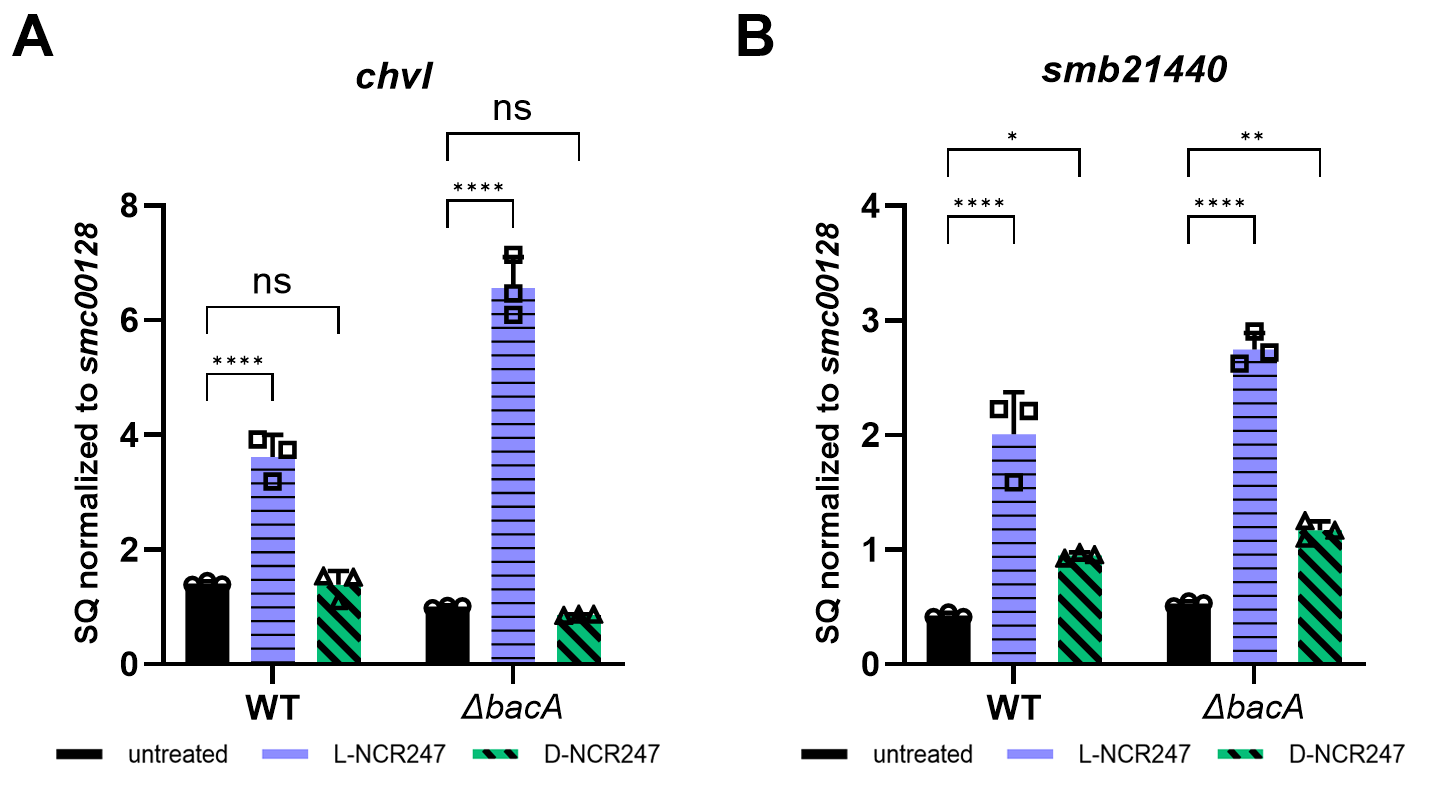

Supplement: S1 Fig — Treatment with 4 µM D-NCR247 shows very little change in expression when compared to untreated cells (Green). In a ∆bacA mutant, there is a significant increase in expression when treated with L-NCR247 when compared to wildtype treated at the same condition. The data are expressed as starting quantities (SQ) of respective mRNAs normalized to the control gene smc00128 and are presented as an average of three technical replicates ± s.d, Two-way analysis of variance (ANOVA) with multiple comparisons was used to calculate p values. *, p ≤ 0.05; **, p ≤ 0.01; ****, p ≤ 0.0001. (TIF) [file pgen.1011892.s001.tif]

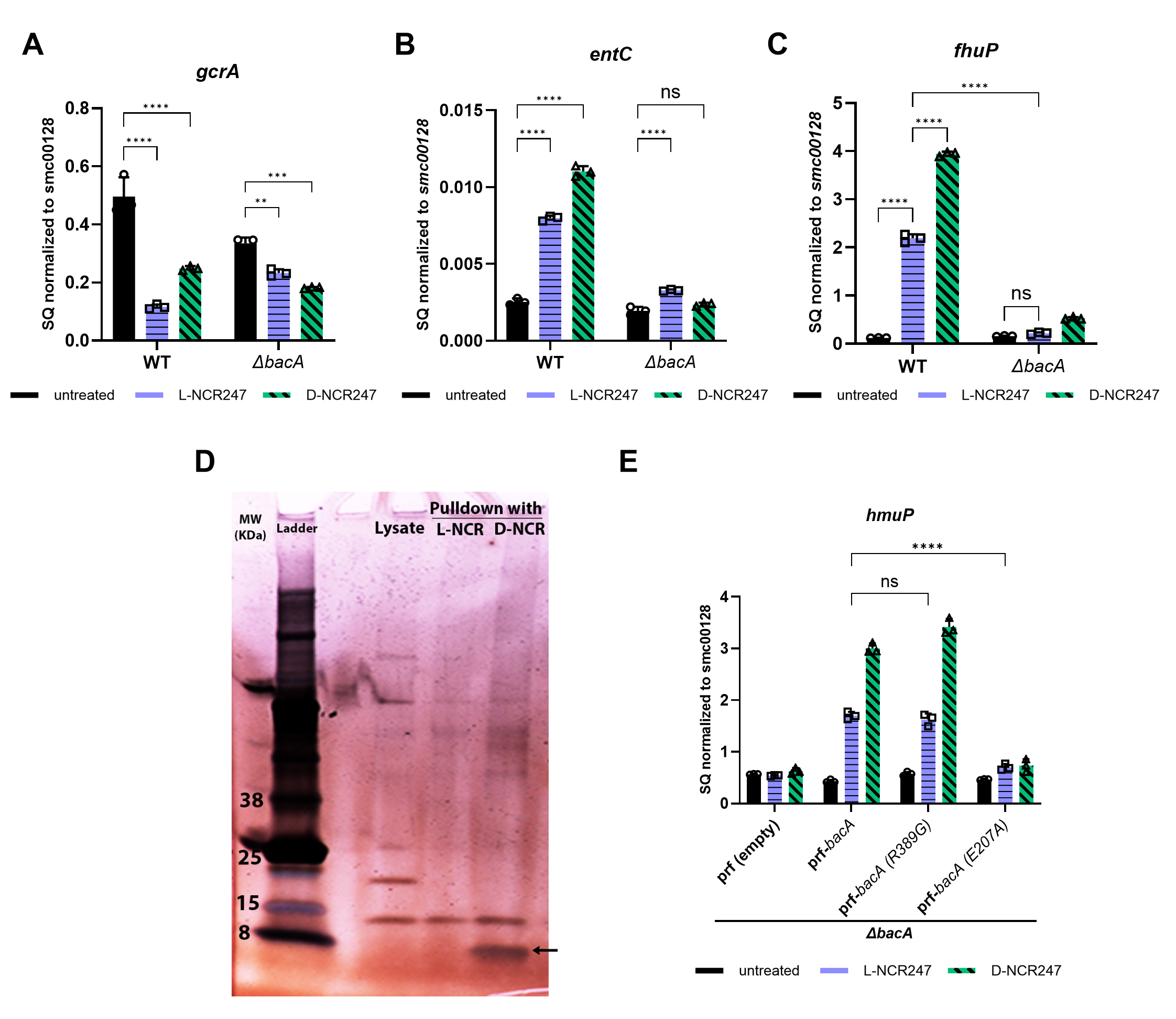

Supplement: S2 Fig — The same treatments resulted in a similar but modest response in a ∆bacA mutant. (B and C) Increase in the expression of the RirA-regulated genes entC and fhuP upon 4 µM L-NCR247 treatment as analyzed by RT-qPCR analysis (blue) as compared to untreated samples (black). Further increase upon D-NCR247 treatment was observed (green). Significant reduction in RirA-regulated gene expression upon both L-NCR247 and D-NCR247 treatment in a ∆bacA mutant when compared to treatments on wildtype is shown. For A, B and C, the data are expressed as starting quantities (SQ) of respective mRNAs normalized to the control gene smc00128 and are presented as an average of three technical replicates ± s.d. (D) Pulldown experiment using streptavidin beads on S. meliloti extracts treated with biotin labelled L- or D-NCR247. * marks the expected size for NCR247. (E) ∆bacA mutant complemented with empty vector shows similar gene expression change (hmuP) in response to peptide treatments as ∆bacA mutant complemented with peptide transport mutant of BacA (prf-bacA(E207A)) as measured by Rt-qPCR analysis indicating the importance of peptide transport. The ∆bacA mutant complemented with BacA defective in VLCFA modification (prf-bacA(R389G)) shows gene expression change like wildtype BacA complemented ∆bacA mutant (prf-bacA). The data are expressed as starting quantities (SQ) of respective mRNAs normalized to the control gene smc00128 and are presented as an average of three technical replicates ± s.d. For A, B, C and E, two-way analysis of variance (ANOVA) with multiple comparisons was used to calculate p values. **, p ≤ 0.01; ***, p ≤ 0.001; ****, p ≤ 0.0001. (TIF) [file pgen.1011892.s002.tif]

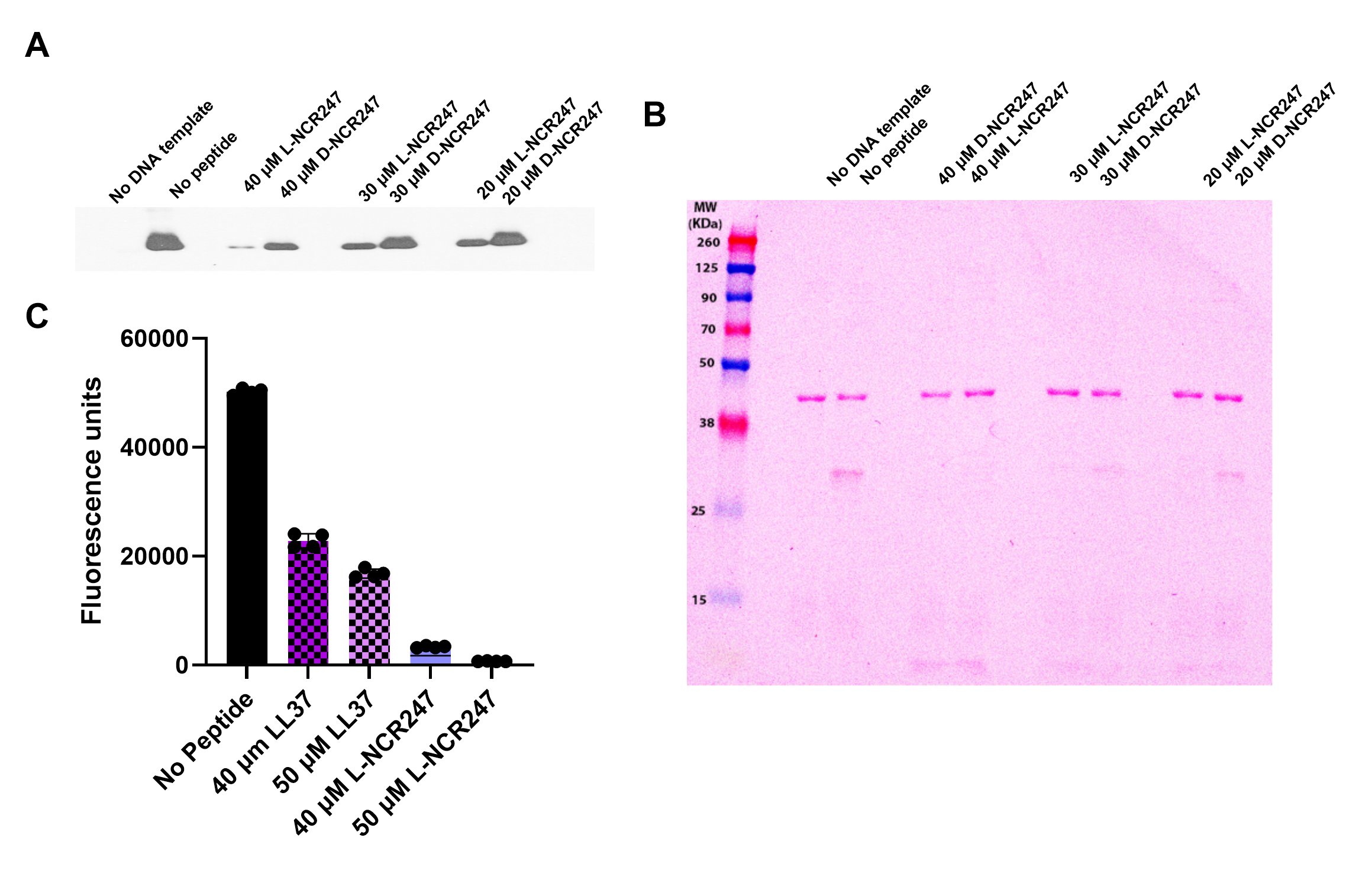

Supplement: S3 Fig — (B) Ponceau stain of the same blot to show loading controls. (C) In vitro translation assay using LL37 with same molar comparison to L-NCR247. Data is presented as the mean of four technical replicates ± s.d. (TIF) [file pgen.1011892.s003.tif]

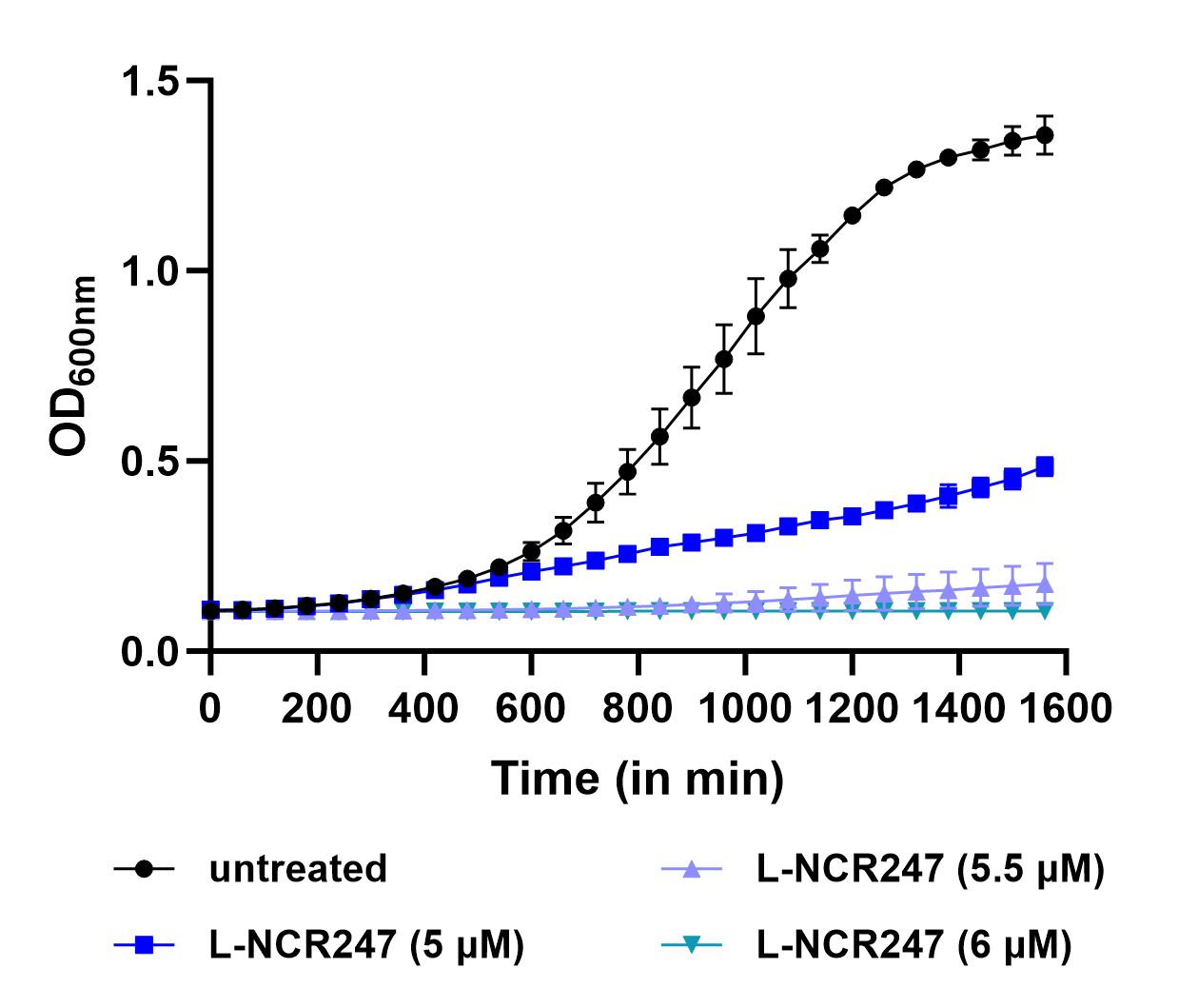

Supplement: S4 Fig — Data is presented as the mean of three biological replicates ± s.d. (TIF) [file pgen.1011892.s004.tif]

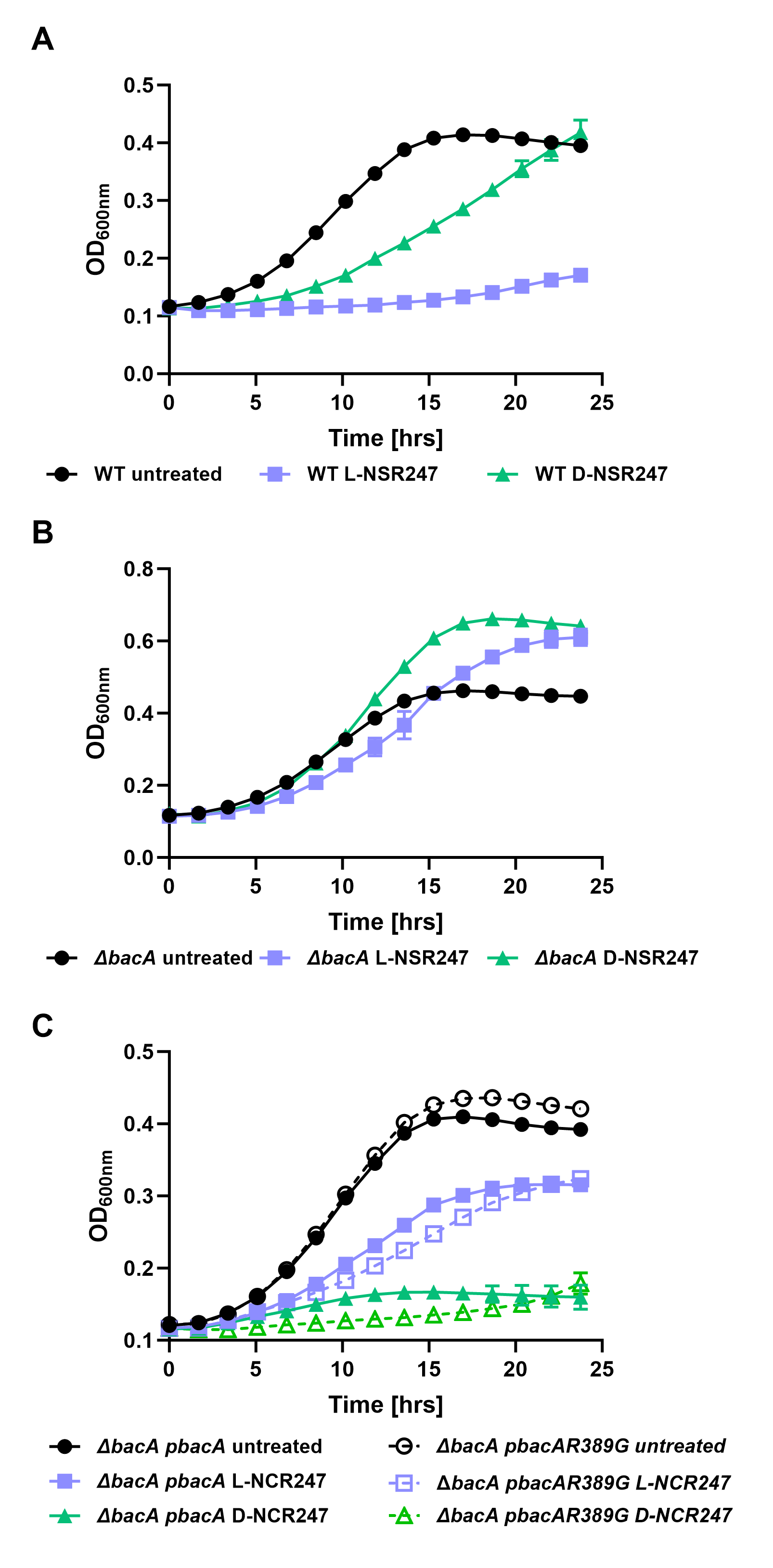

Supplement: S5 Fig — (C) Growth curve analysis over a period of 24 hours on ∆bacA mutant complemented with either wildtype bacA (∆bacA pbacA) or bacA (R389G) ((∆bacA pbacAR389G) upon treatment with 4 µM L –NCR247 (blue) or D-NCR247 (Green). Data is presented as the mean of three biological replicates ± s.d. (TIF) [file pgen.1011892.s005.tif]

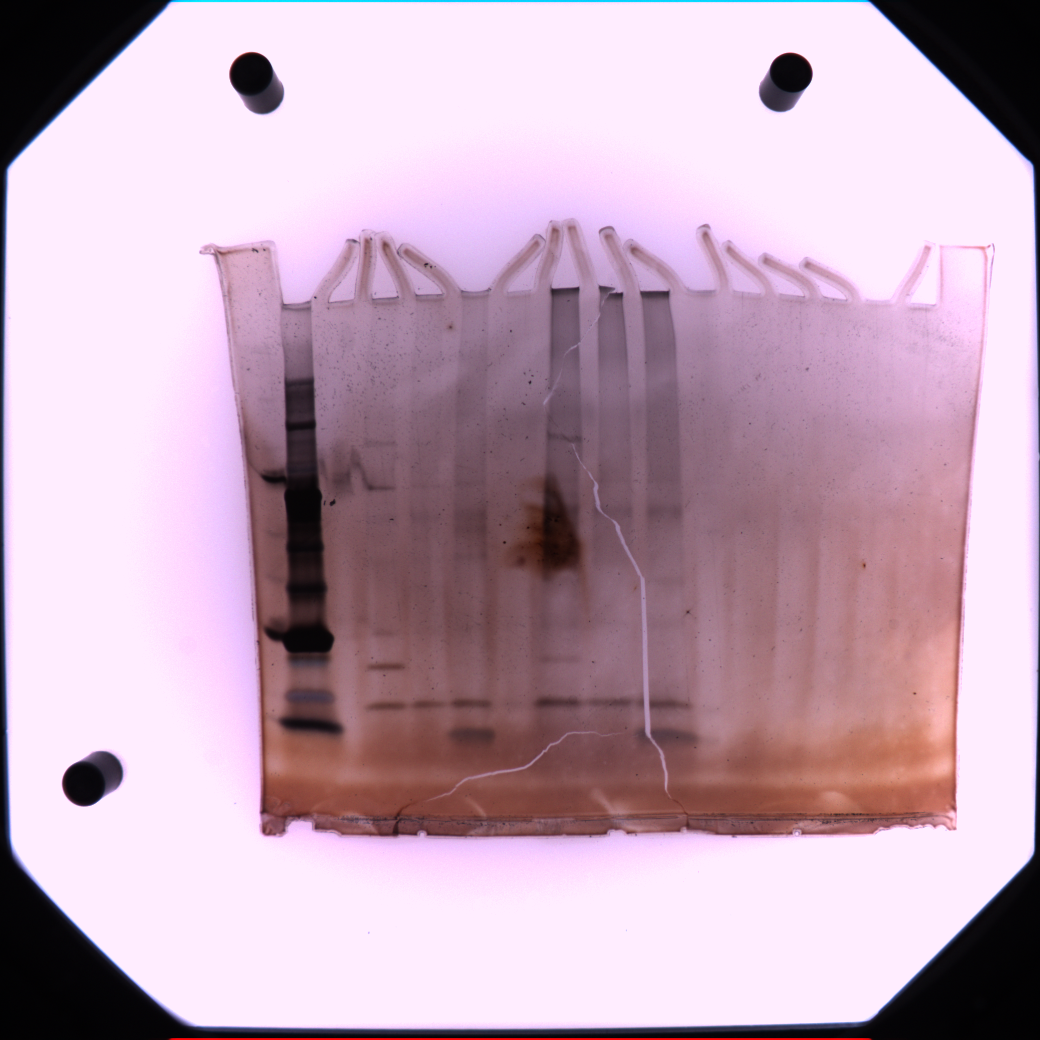

Supplement: S1 Data — (ZIP) [file pgen.1011892.s008.zip › Raw data files/S2 FigD.tif]

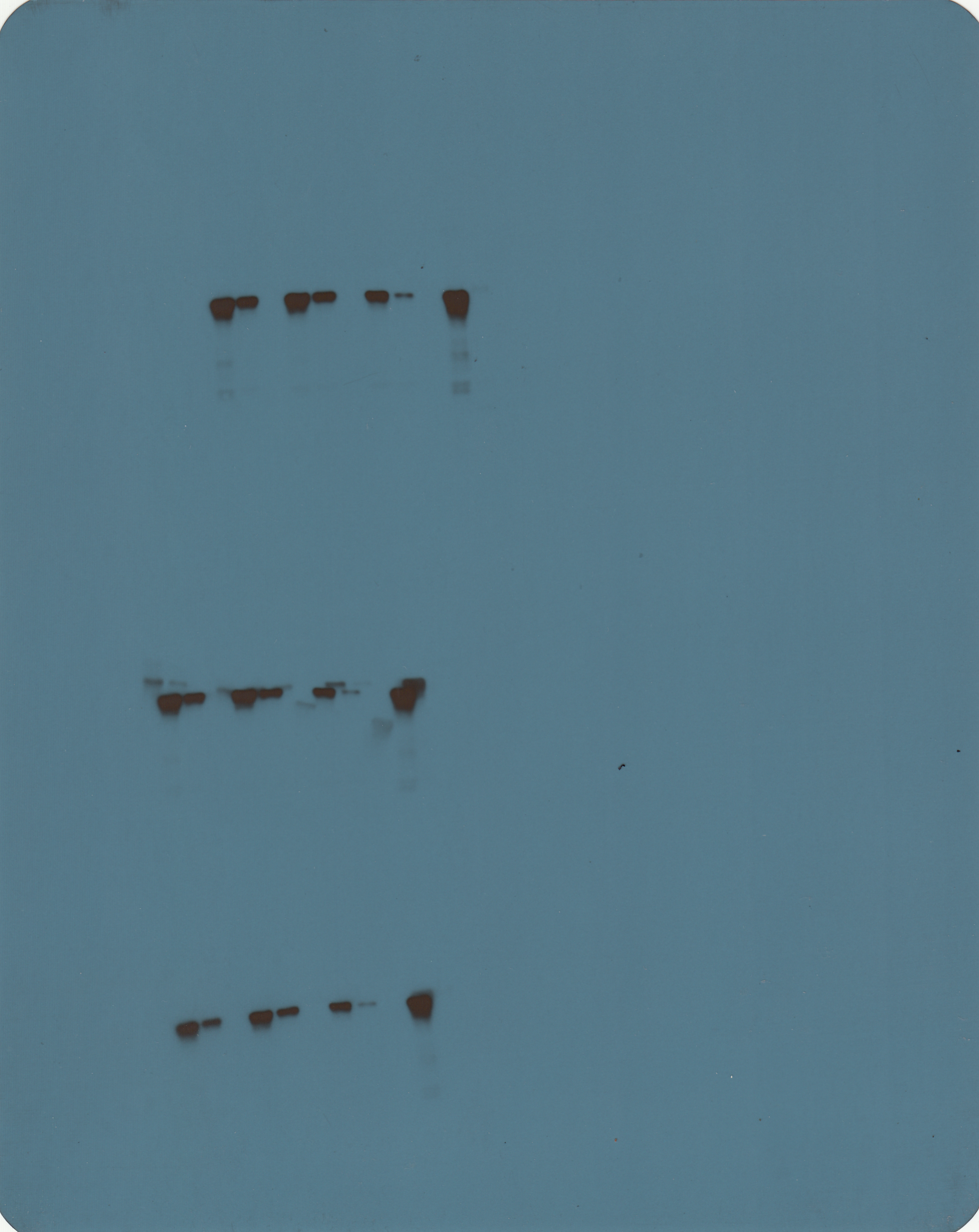

Supplement: S1 Data — (ZIP) [file pgen.1011892.s008.zip › Raw data files/S3 FigA.tif]

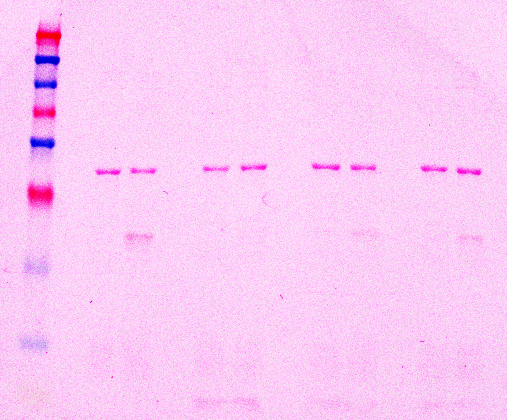

Supplement: S1 Data — (ZIP) [file pgen.1011892.s008.zip › Raw data files/S3 FigB.tif]
