# Supplementary material for: Exploiting peptide chirality and transport to dissect the complex mechanism of action of host peptides on bacteria
Source: PLoS Genet. 2025 Dec 11;21(12):e1011892. doi: 10.1371/journal.pgen.1011892 (PMC12714254; doi:10.1371/journal.pgen.1011892)
Supplement: S1 Table — (DOCX) [file pgen.1011892.s006.docx]

**S1 Table**

**Summary of phenotypes observed with L and D NCR247 treatment in wildtype and *∆bacA* mutant**

| **Phenotype / Response** | **Primary Site of Action** | **Chiral / Achiral** | **BacA dependance** | **Likely Target(s) / Mechanism** |
| --- | --- | --- | --- | --- |
| Higher concentration killing / membrane blebbing | Membrane | Achiral | No | Electrostatic membrane disruption (cationic and amphipathic interaction with membrane) |
| FeuP/FeuQ and ExoS/ChvI regulon activation | Periplasm | Chiral  (L only) | No, hyperactivated in *∆bacA* | Periplasmic sensor kinases FeuQ, ExoS; disulfide containing L- form triggers signaling |
| CtrA regulon repression / cell cycle arrest | Periplasm + Cytoplasm | Partially chiral | modestly enhanced in *∆bacA* | Periplasmic signaling (minimal) + possible cytoplasmic effectors of CtrA pathway |
| Heme sequestration / iron starvation (RirA response) | Cytoplasm | Achiral | Yes, requires BacA import | Direct high affinity binding to heme; triggers iron uptake genes |
| Translation inhibition | Cytoplasm | Partially chiral |  | Ribosomal proteins and/or RNA binding proteins; mix of stereospecific and electrostatic contacts |
| **Additional findings** |  |  |  |  |
| Lethality in *∆bacA* mutant at sublethal L NCR247 | Periplasm | Chiral  (L only) | Yes, lethal in *∆bacA*, suppressed by *∆feuP* | Overstimulation of FeuP signaling cell death |
| Loss of periplasmic lethality with NSR247 | Periplasm | Chiral (dependent on disulfide bonds) | Yes, *∆bacA* is resistant | Disulfide bonds required for toxic periplasmic protein interaction |
| Increased iron response with D-NCR247 | Cytoplasm | Achiral | Yes, requires BacA import | D form more stable in cytoplasm; L form degraded or modified |
| Complementation by BacA homologues (*B. abortus / M. tuberculosis*) | Transport across inner membrane | -- | -- | Conserved function; likely AMP compartmentalization |
