# Supplementary material for: Exploiting peptide chirality and transport to dissect the complex mechanism of action of host peptides on bacteria
Source: PLoS Genet. 2025 Dec 11;21(12):e1011892. doi: 10.1371/journal.pgen.1011892 (PMC12714254; doi:10.1371/journal.pgen.1011892)
Supplement: S2 Table — (DOCX) [file pgen.1011892.s007.docx]

**S2 Table**

**Strains and plasmids used in this study**

| ***Bacterial strain*** | ***Relevant characteristics*** | **Source** |
| --- | --- | --- |
| *Sm*1021 wild type | Smr derivative of SU47 | Meade *et al.*, 1982[1] |
| *Sm*1021 Δ*bacA* | Sm1021, *bacA654*::SpcR | Ferguson *et al.*, 2002 [2] |
| *Sm*1021 Δ*bacA ∆feuP* | Sm1021, *bacA654*::SpcR with *feuP* deletion | This study |

| **Plasmid** | ***Relevant characteristics*** | **Source** |
| --- | --- | --- |
| pRF771 | RK2 derivative Ptrp expression vector  *tetR* | Wells DH *et al.*, 2002 [3] |
| p*SmbacA* | pRF771 carrying the *S. meliloti bacA* gene under control of a constitutive Ptrp promoter, *tetR* | Arnold *et al*., 2025 [4] |
| p*bacAR389G* | pRF771 carrying the *S. meliloti bacA* gene with R389G mutation under control of a constitutive Ptrp promoter,  *tetR* | This Study |
| p*bacAE207A* | pRF771 carrying the *S. meliloti bacA* gene with E207A mutation under control of a constitutive Ptrp promoter,  *tetR* | This Study |
| p*MtbacA* | pRF771 carrying the *M. tuberculosis* *bacA* gene under control of a constitutive Ptrp promoter,  *tetR* | This Study |
| p*BabacA* | pRF771 carrying the *B. abortus bacA* gene under control of a constitutive Ptrp promoter,  *tetR* | This Study |
| pJG206 | Gent SacB selection with FeuP flanks | Rebecca Carolyn et al, 2011 [5] |

**Supplementary References:**

1. Ruvkun GB, Long SR, Meade HM, van den Bos RC, Ausubel FM (1982) ISRm1: A Rhizobium meliloti insertion sequence that transposes preferentially into nitrogen fixation genes. J Mol Appl Genet 1: 405-418.

2. Ferguson GP, Roop RM, 2nd, Walker GC (2002) Deficiency of a Sinorhizobium meliloti BacA mutant in alfalfa symbiosis correlates with alteration of the cell envelope. J Bacteriol 184: 5625-5632.

3. Wells DH, Long SR (2002) The Sinorhizobium meliloti stringent response affects multiple aspects of symbiosis. Mol Microbiol 43: 1115-1127.

4. Arnold MFF, Sankari S, Deutsch M, Gruber CC, Guerra-Garcia FJ, Walker GC (2025) The BacA(SbmA) Importer of Symbiotically Important Legume Nodule Cysteine-Rich Peptides: Insights into Protein Architecture, Function, and Evolutionary Implications bioRxiv 2025.09.17.676847.

5. Carlyon RE, Ryther JL, VanYperen RD, Griffitts JS (2010) FeuN, a novel modulator of two-component signalling identified in Sinorhizobium meliloti. Mol Microbiol 77: 170-182.
